# 

**Published:** 1892-01

**Authors:** 


### LIST OF CONTRIBUTORS TO VOLUME XIII.

Allan, Geo. S., D.D.S.
Atkinson, Charles B., D.D.S.
Bigelow, Lyman F., D.M.D.
Blaisdell, Edwin C., D.M.D.
Bonwill, W. G. A., D.D.S.
Briggs, Chas. P., M.D., D.D.S.
Bryan, L. C., D.D.S.
Buck, E. J., D.D.S.
Carr, William, D.D.S.
Chase, Henry S., M.D., D.D.S.
Chase, R. M., M.D., D.D.S.
Cooke, Wm. P., D.M.D.
Davenport, Isaac B., M.D., D.D.S.
De Trey, E., D.D.S.
Dunn, Wm., Jr., D.D.S.
Eddy, Forrest G., D.M.D.
Elliott, A. V., D.D.S.
Garretson, J. E., M.D., D.D.S.
Gerrish, C. H., D.D.S.
Grey, Richardson, M.D.
Hamilton, H. F., D.D.S.
Harker, Charles, D.D.S.
Head, Joseph, M.D., D.D.S.
Heitzmann, C., M.D.
Hugenschmidt, A. C., M.D., D.D.S.
Hugo, L. C. F., D.D.S.
Jack, Louis, D.D.S.
Kelley, Henry A., D.M.D.
Kirk, Edward C., D.D.S.
Magill, W. E., D.D.S.
Marshall, John S., M.D.
Maxfield, George A., D.D.S.
McIntosh, L. D., M.D., D.D.S.
Mills, G. A., D.D.S.
Mitchell, W., D.D.S.
Moriarty, P. W., D.M.D.
Osmun, J. Allen, D.D.S.
Ottolengui, Rodrigues, D.M.D.
Palmer, S. B., D.D.S.
Patterson, A. Bethune, M.D.
Patton, W. R., D.D.S.
Peabody, D. D., D.D.S.
Peeso, Frederic A., D.D.S.
Peirce, C. N., D.D.S.
Porter, J. M., D.D.S.
Register, J. E., D.D.S.
Richmond, C. M., D.D.S.
Rollins, W. H., D.D.S.
Roy, F. A., M.D., D.D.S.
Seabury, Frederick N., D.D.S.
Stanton, Jere. E., M.D., D.M.D.
Stebbins, E. H., D.D.S.
Stellwagen, Thomas C., M.D., D.D.S.
Stevens, Edgar F., D.M.D.
Sudduth, W. Xavier, A.M., M.D., D.D.S.
Taft, Chas. H., A.B., D.M.D.
Trueman, William H., D.D.S.
Tucker, E. G., D.D.S.
Warren, G. W., D.D.S.
Weld, G. W., M.D., D.D.S.
Westlake, Albert, D.D.S.
White, E. B., D.D.S.
Williams, N. W., D.D.S.
Woodward, Wm. A., D.D.S.

SUBSCRIBE FOR THE

### International Dental Journal

**The Organ of the Profession,**

PUBLISHED BY THE

INTERNATIONAL DENTAL PUBLICATION COMPANY:

**LOUIS JACK,** *President,* Philadelphia.
**BENJAMIN LORD,** *Vice-President,* New York.
**C. N. PEIRCE,** *Treasurer,* Philadelphia.
**GEO. S. ALLAN,** *Secretary,* 51 W. 37th St., New York

*PUBLICATION OFFICE:*

716 Filbert Street, Philadelphia, Pa.

**FOREIGN CORRESPONDENTS:**

Dr. ARKÖVY, Budapesth, Hungary.
W. GEO. BEERS, D.D.S., Ed. Dominion Dental Journal, Montreal.
L. C. BRYAN, D.D.S., Basel.
ALFRED BURNE, D.D.S., Sydney, New South Wales.
F. BUSCH, M.D., Berlin.
GEORGE CUNNINGHAM, Cambridge, Eng.
I. B. DAVENPORT, D.D.S., Paris.
PAUL DUBOIS, Ed. L'Odontologie, Paris.
WILLIAM DUNN, Jr., L.D.S., Florence.
A. H. EDWARDS, D.D.S., Madrid.
W. ST. GEORGE ELLIOTT, M.D., D.D.S., Lon.
OSWALD FERGUS, D.D.S., Glasgow, Scotland.
ALPHONSE FEUCHEL, Zahnarzt, St. Petersburg.
C. R. FLETCHER, D.D.S., Sao Paulo, Brazil.
WALTER HARRISON, L.D.S., D.M.D., Brighton, Eng.
ROBERT S. IVY, D.D.S., Shanghai, China.
N. S. JENKINS, D.D.S., Dresden.
F. KLAPPROTH, Zahnarzt, St. Petersburg.
L. KOLBE, " " "
Dr. GEO. P. E. KUHN, Paris.
W. BOWMAN MACLEOD, L.D.S., Edinburgh.
W. D. MILLER, Ph. D., M.D., D.D.S., Berlin.
WILLIAM SACHS, D.D.S., Breslau.
J. G. VAN MARTER, B.A., D.D.S., Rome.
J. M. WHITNEY, M.D., D.D.S., Honnolula.
LUDWIG WARNEKROS, Zahnarzt, Berlin.
LUDW. ADOLPH WEIL, M.D., Munich.
J. COWAN WOODBURN, M.D., Glasgow.

STOCKHOLDERS:

Frank Abbott,
Geo. S. Allan,
W. W. Allport,
R. R. Andrews,
H. A. Baker,
A. E. Baldwin,
W. C. Barrett,
A. P. Beale,
S. T. Beale, Jr.,
C. S. Beck,
G. V. Black,
C. F. W. Bödecker,
E. A. Bogue,
W. G. A. Bonwill,
C. A. Brackett,
Thomas Bradley,
Edward C. Briggs,
Albert H. Brockway
A. E. Brown,
Wm. Carr,
Geo. H. Chance,
G. F. Cheney,
Dwight M. Clapp,
John D. Codman,
Chas. D. Cook,
J. N. Crouse,
Edw. T. Darby,
H. S. Draper,
Wm. H. Dwinelle,
A. R. Eaton,
Chas. J. Essig,
J. N. Farrar,
T. Fillebrown,
W. B. Finney,
T. A. Fletcher,
M. W. Foster,
Chas. E. Francis,
E. C. Fundenberg,
W. F. Fundenberg,
W. H. Fundenberg,
E. S. Gaylord,
J. P. Geran,
A. H. Gilson,
S. H. Guilford,
John I. Hart,
O. E. Hill,
J. F. P. Hodson,
J. Morgan Howe,
Robert Huey,
A. O. Hunt,
Louis Jack,
V. H. Jackson
C. R. Jefferis,
C. A. Kingsbury,
C. R. E. Koch,
W. T. La Roche,
W. K. Leech,
F. A. Levy,
Wilbur F. Litch,
J. Bond Littig,
Benjamin Lord,
Geo. W. Lovejoy (Canada),
John S. Marshall,
H. J. McKellops,
Charles McManus,
James McManus,
Dan'l Neal McQuillan,
Horatio C. Meriam,
W. D. Miller (Berlin),
W. E. Page,
S. B. Palmer,
C. B. Parker,
D. D. Peabody,
C. N. Peirce,
S. G. Perry,
W. A. Phreaner,
Chas. E. Pike,
W. E. Pinkham,
W. H. Potter,
Chas. P. Pruyn,
H. C. Register,
F. A. Roy,
Z. T. Sailer,
L. D. Shepard,
Geo. R. Shidle,
Eugene H. Smith,
B. Holly Smith,
H. A. Smith,
S. G. Stevens,
W. X. Sudduth,
Eugene S. Talbott,
Ambler Tees,
J. D. Thomas,
James Truman,
Wm. H. Trueman,
W. W. Walker,
I. F. Wardwell,
G. W. Warren,
T. S. Waters,
Geo. W. Weld,
Julius G. W. Werner,
Jacob L. Williams,
R. B. Winder,
C. A. Woodward,
J. A. Woodward,
W. A. Woodward,
W. J. Younger.

***Subscription Price, $2.50 per Year.***

